# BRAF-activated ARSI suppressed EREG-mediated ferroptosis to promote BRAF^V600E^ (mutant) papillary thyroid carcinoma progression and sorafenib resistance

**DOI:** 10.7150/ijbs.99423

**Published:** 2025-01-01

**Authors:** Xing Chen, Xiang Chen, Wenjun Xie, Hua Ge, Hongyan He, Ailong Zhang, Junjie Zheng

**Affiliations:** 1 Department of Thyroid and Hernia Surgery, Shengli Clinical Medical College of Fujian Medical University, Fujian Provincial Hospital, Fuzhou University Affiliated Provincial Hospital, Fuzhou City, Fujian Province 350001, China.; 2 Department of Ultrasonography, Shengli Clinical Medical College of Fujian Medical University, Fujian Provincial Hospital, Fuzhou University Affiliated Provincial Hospital, Fuzhou City, Fujian Province 350001, China.; 3 Department of Nuclear Medicine, Shengli Clinical Medical College of Fujian Medical University, Fujian Provincial Hospital, Fuzhou University Affiliated Provincial Hospital, Fuzhou City, Fujian Province 350001, China.; 4 Department of Pathology, Shengli Clinical Medical College of Fujian Medical University, Fujian Provincial Hospital, Fuzhou University Affiliated Provincial Hospital, Fuzhou City, Fujian Province 350001, China.

**Keywords:** BRAF, PTC, ferroptosis, ARSI, EREG

## Abstract

Papillary thyroid carcinoma (PTC) is the most common type of thyroid cancer, and patients with the BRAF^V600E^ mutation often exhibit aggressive tumor behavior. Here, we identified Arylsulfatase I (ARSI) as a gene whose expression was significantly upregulated in BRAF^V600E^ PTC and was associated with poor prognosis. High ARSI expression correlated with advanced disease stage, BRAF mutation, and worse overall survival in PTC patients. Functional studies revealed that ARSI promoted the tumor growth, cell migration, and epithelial-mesenchymal transition (EMT) of BRAF^V600E^ PTC cells *in vitro*. *In vivo* studies confirmed that ARSI suppression inhibited tumor growth and metastasis in mouse models of PTC. Mechanistically, ARSI knockdown triggered ferroptosis in BRAF^V600E^-mutant PTC cells and sensitized PTC cells to sorafenib-induced ferroptosis. Epiregulin (EREG) was identified as a downstream target of ARSI and is regulated by STAT3 transcriptional activation. EREG overexpression rescued the ferroptosis resistance and malignant phenotypes induced by ARSI knockdown in BRAF^V600E^-mutant PTC cells. Finally, we constructed a prognostic signature and diagnostic model based on ARSI and EREG expression data, which demonstrated high predictive value for identifying high-risk PTC patients with the BRAF^V600E^ mutation. Our study highlights the critical role of ARSI in promoting aggressive phenotypes and therapeutic resistance in BRAF^V600E^ PTC through ferroptosis regulation. Targeting the ARSI-EREG axis may offer novel therapeutic avenues for improving outcomes in BRAF^V600E^ PTC patients.

## Introduction

Thyroid cancer (TC) is the most common endocrine malignancy and ranks ninth in cancer incidence worldwide^1^; its incidence has increased in recent years. Approximately 80-85% of cases are of the papillary thyroid cancer (PTC) subtype, which is known for its favorable prognosis, with a 10-year survival rate of 80-90%^2^, ^3^. However, studies indicate that PTC patients have an approximately 30% recurrence rate within 5 years, impeding clinical treatment and survival for some patients^4^-^6^. The BRAF^V600E^ mutation is the most driver mutation in PTC, with extensive research confirming its association with PTC invasiveness and iodine-resistant phenotypes^7^-^9^. BRAF is a cytoplasmic serine-threonine protein kinase, and the BRAF^V600E^ mutation causes constitutive activation of the MAPK pathway, leading to enhanced cell proliferation and malignant transformation, thus promoting the development of malignant tumors^10^-^12^. Our previous research has shown that Wilms' tumor 1 (WT1) promotes the malignant progression of PTC in cells harboring the BRAF^V600E^ mutation through various cellular biological processes^13^. However, other molecular mechanisms involved in PTC and the role of the BRAF^V600E^ mutation in PTC progression and development remain to be further elucidated.

Arylsulfatase I (ARSI), a member of the sulfatase gene family, is primarily expressed in embryonic tissues^14^, ^15^. Unlike its counterparts, ARSI functions as a secreted protein in the extracellular environment, undergoing rapid degradation in either the endoplasmic reticulum (ER) or the medium. Excess ARSI accumulates within the ER in a denatured state, consequently leading to SUMF1 degradation^16^. Studies evaluating ARSI expression across various human tissues and cancer cell lines have revealed its predominant presence in tissue remodeling during both tumor progression and embryonic development^17^. Despite extensive research on the ARSI gene, its precise involvement in PTC tumorigenesis has not been fully elucidated.

Ferroptosis is an iron-dependent programmed cell death process associated with the accumulation of reactive oxygen species (ROS) and increased lipid peroxidation^18^, ^19^. When the metabolism of lipid peroxides catalyzed by glutathione peroxidase 4 (GPX4) and the intracellular antioxidant glutathione (GSH) decreases, lipids are oxidized by Fe^2+^ in a Fenton-like manner, ultimately resulting in an increase in intracellular lipid reactive oxygen species (ROS) and promoting ferroptosis^20^, ^21^. Dysregulation of ferroptosis is reportedly associated with several cancers, including PTC, and its crucial role in inhibiting tumor development is widely accepted. In recent years, several ferroptosis-related genes have been shown to play a critical role in predicting the prognosis of PTC patients^22^-^24^. However, the specific mechanisms of ferroptosis in BRAF^V600E^ PTC are currently not well understood.

Herein, we found increased ARSI expression in BRAF^V600E^ PTC, which is associated with malignant phenotypes and a poor prognosis. Inhibition of ARSI *in vitro* and *in vivo* increased sorafenib-induced ferroptosis in PTC cells. Mechanistically, ARSI-mediated STAT3 phosphorylation is required for the transcription of EREG, which is involved in BRAF^V600E^-induced tumorigenesis and ferroptosis resistance. Importantly, our work suggests a potential therapeutic strategy for enhancing chemosensitivity in BRAF^V600E^ PTC patients.

## Materials and methods

### Data acquisition and differentially expressed gene (DEG) screening

The transcriptome data and corresponding clinical signatures of patients, which included 58 normal thyroid and 500 PTC patients, were downloaded from the TCGA database. We utilized the limma package in the R statistical software to detect DEGs in the TCGA datasets, employing a log2 (fold change) threshold >1.0 and a significance threshold of p < 0.05.

### Functional enrichment

Gene Ontology (GO) enrichment analysis and Kyoto Encyclopedia of Genes and Genomes (KEGG) analysis were performed using the 'clusterProfiler' R package (http://www.bioconductor.org/packages/release/bioc/html/clusterProfiler.html). Terms with P<0.05 were considered significantly enriched.

### Cell culture and reagents

PTC cell lines, including IHH4 and BCPAP, were procured from the American Type Culture Collection (ATCC) (Manassas, VA, USA). For culture, IHH4 cells were maintained in a mixture of DMEM and RPMI 1640 in equal proportions supplemented with 2 mM L-glutamine, 10% fetal bovine serum (FBS), and 100 U/ml penicillin. BCPAP cells were cultured in FK12 medium supplemented with 10% FBS and 100 U/mL penicillin. Both cell lines were incubated at 37 °C in a 5% CO_2_ atmosphere with 95% atmosphere at constant temperature.

### Western blotting

The cells were washed with cold PBS, and total proteins were extracted with RIPA buffer (Beyotime Biotechnology). These proteins were subsequently separated by size through sodium dodecyl sulfate‒polyacrylamide gel electrophoresis (SDS‒PAGE). The proteins were then transferred from the gel to a membrane, which preserves their spatial distribution. The blots were blocked with 5% milk in PBS-T (0.01% Tween-20 in phosphate-buffered saline) and then incubated overnight at 4 °C with primary antibodies. Following primary antibody binding, HRP-conjugated secondary antibodies were applied at room temperature for 2 h. The intensity of the signal was scanned using an OdysseyIR scanner (Li-CORBiosciences, NE) and quantified using ImageJ software.

### Quantitative real-time PCR (RT-qPCR)

Total RNA was extracted from the cultivated cells using TRIzol reagent (Invitrogen, CA, USA), and the RNA was reverse transcribed into cDNA. RT‒qPCR analysis was performed on 100 ng of total RNA using an iTaq Universal SYBR Green One-Step Kit (Bio-Rad, CA) on a CFX96 instrument (Bio-Rad, CA). The mRNA levels were normalized to those of β-actin. The primers used for RT‒qPCR analysis are listed in Supplementary Table S1.

### Chromatin immunoprecipitation (ChIP)

ChIP assays were performed essentially as described previously^25^. In brief, after immunoprecipitation using a rabbit polyclonal anti-STAT3 antibody and DNA extraction, quantitative PCR was performed using primers specific for the promoter region of mouse EREG genes (primer sequences are presented in Supplementary Table S2).

### Cell viability assay

Cells (3000 to 4000/well) were plated in 96-well white-edged plates. After 24 h of culture, the cells were treated with different concentrations of inhibitors or DMSO for 48 h. A viability assay was performed using a Cell Counting Kit-8 (CCK-8; C0039; Beyotime, Shanghai, China). The absorbance was determined at a wavelength of 450 nm on a GloMax96 microplate reader.

### Lipid peroxidation assay

A lipid peroxidation staining assay was performed using BODIPY 581/591 C11 dye (Invitrogen, D3861) as previously described^26^. After the cells were incubated with the dye in serum-free medium at 37 °C for 30 min, the cells were washed with HBSS. Lipid ROS levels were analyzed with FlowJo software (version 10.0). At least 10,000 cells were collected per sample.

### Wound healing assay

A total of 5 × 10^5^ cells were plated in each well of a 12-well plate and allowed to reach confluence. Subsequently, a scratch was made in each culture using a pipette tip. After 48 h, precise measurements of the wound were taken to determine wound closure, calculated as (initial wound width - wound width at 48 h)/initial wound width × 100%. The cultures were observed and imaged using an inverted phase-contrast microscope.

### Ethynyldeoxyuridine (EdU) staining

To evaluate the fraction of DNA-replicating cells, which is indicative of cell proliferation status, EdU staining was performed using the Cell-Light EdU Apollo488 *In Vitro* Imaging Kit, following the guidelines provided by the manufacturer (C10310-3, RiboBio, Guangzhou, China).

### Xenograft mouse model

Four-week-old female nude mice were maintained under special pathogen-free (SPF) conditions and randomly divided into different groups. A total of 2 × 10^6^ ARSI knockdown or control PTC cells were subcutaneously injected into the backs of nude mice. Tumor length (L) and width (W) were measured every 3 days, and tumor volume (V) was calculated as follows: (3.14 × L × W2)/6. Finally, all the mice were sacrificed and dissected immediately to measure the tumor weights.

### Statistical analysis

Statistical analysis of the experimental results was performed using GraphPad Prism software (version 6.0) and R software (version 4.0.3). Differences between two groups were analyzed using Student's t test, and all the results are shown as the means ± SEMs. The statistical significance was set at P < 0.05, and each experiment was repeated three times.

## Results

### ARSI is upregulated in BRAF^V600E^ PTC samples and correlates with poor prognosis

**Figure 1:**
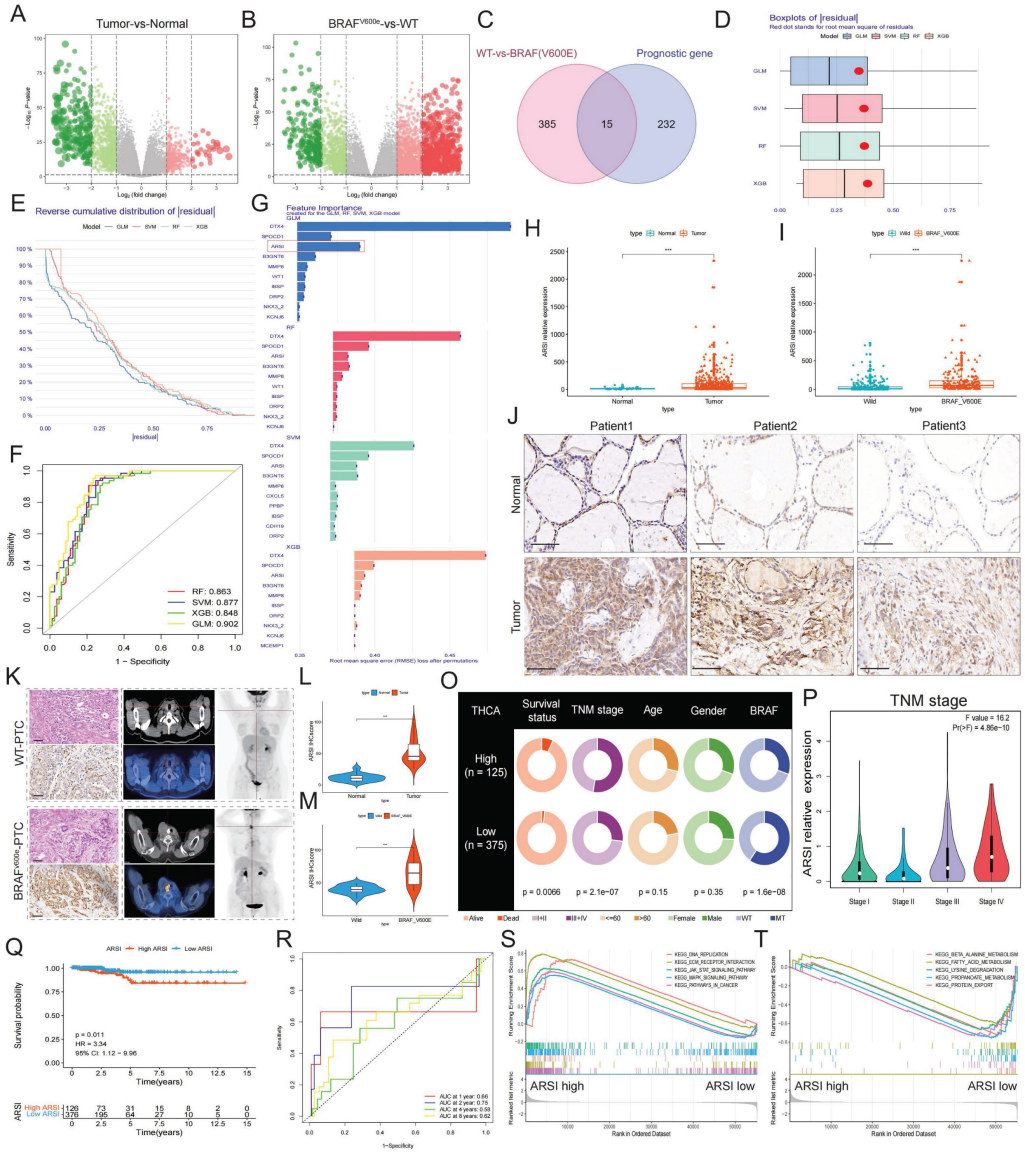
** ARSI is upregulated in BRAF-mutated PTC tissue and predicts poor prognosis in PTC patients. A.** Screening for DEGs between PTC tumor tissues and normal tissues from the TCGA-THCA cohort. **B.** Screening for DEGs based on the BRAF V600E mutation status in PTC patients. **C.** Venn diagram illustrating the overlap of BRAF V600E mutation-related genes and prognostic genes in the PTC cohort. **D.** Boxplots of ∣residual∣ in the GLM, support vector machine (SVM), random forest (RF) and XGB models. The red dots in the “Boxplots of residuals” represent the mean values of the residuals of all the samples.** E.** Cumulative distributions of the GLM, SVM, RF and XGB models. **F.** ROC curve and AUC values of the GLM, SVM, RF and XGB models. **G.** The important features in the GLM, SVM, RF and XGB models. **H.** ARSI levels in PTC and noncancer tissues.** I.** ARSI expression based on the BRAF mutation genotype in the THCA cohort. **J.** Immunohistochemical staining of the ARSI protein based on the BRAF mutation status in PTC samples. **K.** Representative IHC and PET-CT scans of PTC patients. **L, M.** IHC score. **N.** Assessment of the relationship between ARSI expression and clinical parameters. **O.** Correlation between ARSI expression and clinical stage in PTC patients. **P.** Kaplan-Meier analysis of overall survival (OS) based on ARSI expression in PTC patients with BRAF mutations. **Q.** Time-dependent ROC analysis for OS prediction in the THCA cohort. **R, S.** Representative KEGG pathways associated with ARSI expression identified via GSEA.

We screened the TCGA-THCA cohort, including the clinical characteristics of 510 PTC patients, to identify pivotal genes correlated with the prognosis of PTC patients (Fig. 1A). PTC samples were further classified by BRAFV600E mutation status to identify DEGs affected by BRAF (Fig. 1B). Fifteen core genes associated with poor prognosis and BRAF^V600E^ mutation status were displayed using a Venn diagram (Fig. 1C). To further identify subtype-specific genes with high diagnostic value, we established four proven machine learning models [the generalized linear model (GLM), support vector machine model (SVM), random forest model (RF) and eXtreme Gradient Boosting (XGB)] based on the 15 hub regulators. The GLM machine learning model presented a relatively lower residual (Figs. 1D, E). Moreover, we evaluated the discriminative performance of the four machine learning algorithms in the testing set by calculating receiver operating characteristic (ROC) curves based on 5-fold cross-validation. The GLM machine learning model displayed the highest area under the ROC curve (AUC) (GLM, AUC = 0.902; SVM, AUC = 0.877; RF, AUC =0.863; XGB, AUC = 0.848; Fig. 1F). Subsequently, the top 10 important feature variables of each model were ranked according to the root mean square error (RMSE) (Fig. 1G). Overall, combined with these results, the GLM was demonstrated to best distinguish patients with different clusters. We investigated the relationship between ARSI expression and clinicopathological features in the TCGA cohort. High ARSI expression was associated with tumor tissue and BRAF mutation (Figs. 1H, I). The overexpression of the ARSI protein in PTC was also confirmed by IHC (Fig. 1J). PET-CT imaging of BRAF^V600E^ PTC patients revealed a heavy tumor load, and immunohistochemistry results suggested increased Ki-67 expression (Figs. 1K-M). We also found that ARSI expression was significantly linked to survival status, TNM stage and BRAF mutation (Fig. 1N), and the expression of ARSI was greater in patients with advanced TNM stage (Fig. 1O). Kaplan-Meier survival analysis was performed on the TCGA dataset to evaluate the relationship between overall survival (OS) and the expression of ARSI. High ARSI expression correlated with significantly poorer outcomes (Figs. 1P, Q). GSEA also showed that tumor progression-related signaling pathways, including the ECM_receptor_interaction and JAK_STAT_signaling pathways, were closely associated with ARSI expression in PTC (Fig. 1R, S).

### ARSI promotes the growth, migration and epithelial-mesenchymal transition process oof BRAF^V600E^ PTC cells *in vitro*

**Figure 2:**
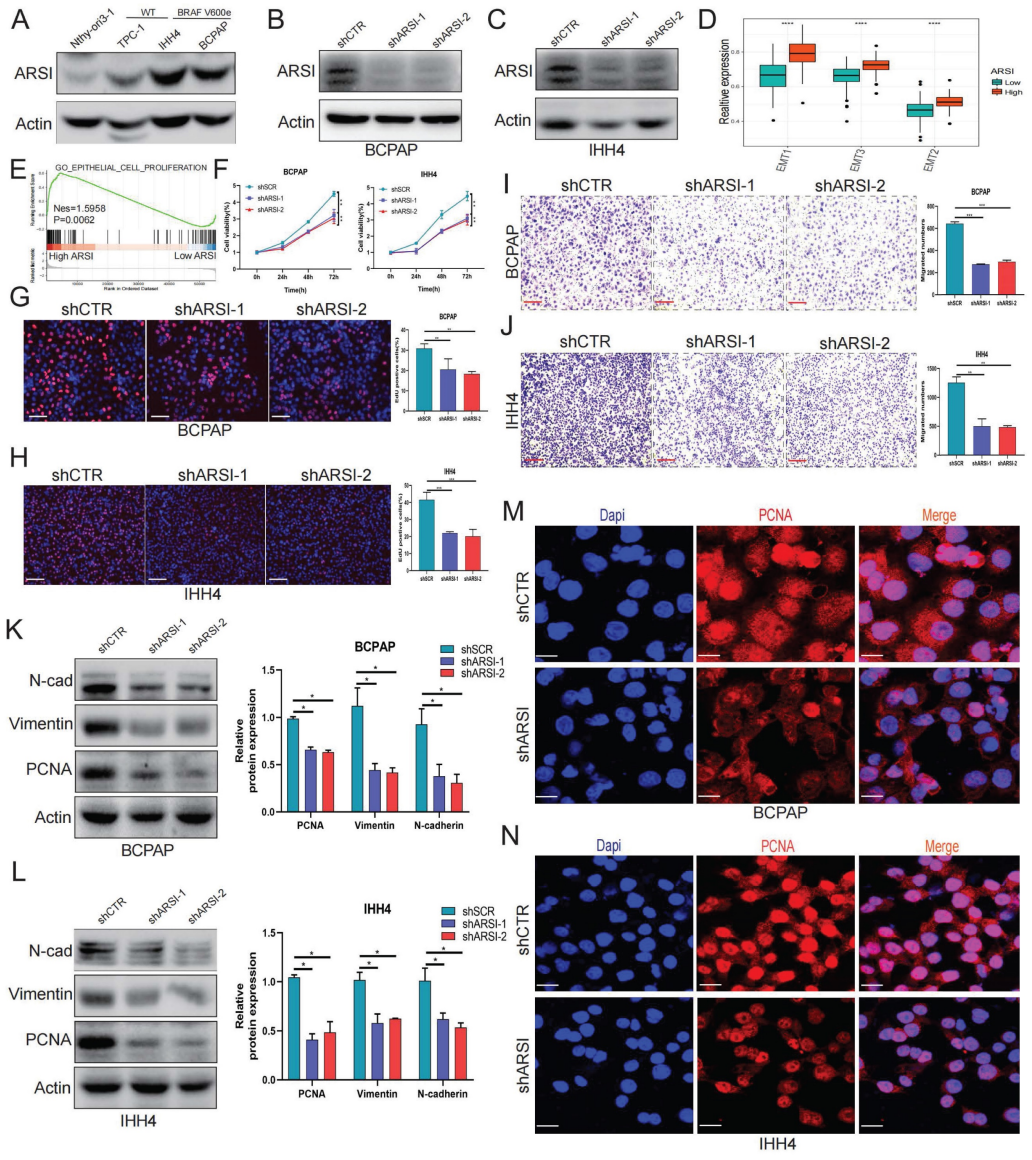
** ARSI promotes the growth, migration and epithelial-mesenchymal transition process oof BRAF^V600E^ PTC cells. A.** Representative immunoblot for cell lysates from BRAF-mutant and wild-type PTC cells. **B,C.** Representative immunoblots showing the knockdown efficiency of ARSI in BCPAP (B) and IHH4 (C) cells.** D.** Box plots of EMT signature scores in the low- and high-ARSI expression groups.** E.** GSEA of enriched genes related to epithelial-mesenchymal proliferation in the high-ARSI expression group. **F.** A CCK8 assay to detect the effect of ARSI knockdown on cell proliferation. **G, H.** EdU assays to detect the effect of ARSI knockdown on cell proliferation. BCPAP (G), IHH4 (H).** I, J.** Wound healing assay to detect the effect of ARSI knockdown on cell migration. BCPAP (I), IHH4 (J). **K, L.** Representative immunoblots of PCNA, N-Cad and vimentin in BCPAP (K) and IHH4 (L) cells after ARSI knockdown.** M, N.** Immunofluorescence staining was used to detect the expression of PCNA in the indicated cells. BCPAP (M), IHH4 (N). The data are expressed as the mean±SE; * p < 0.05, ** p < 0.01, and *** p < 0.001.

As demonstrated above, mutant BRAF was positively associated with ARSI expression in PTC patients, and compared with wild-type BRAF cells, BRAF-mutant PTC cells also displayed significantly elevated ARSI expression (Fig. 2A). We then established stable cell lines expressing ARSI-shRNA via lentiviral infection using BRAF-mutant PTC cells (including BCPAP cells and IHH4 cells) (Figs. 2B, C). GSVA and GSEA revealed that ARSI expression was positively correlated with epithelial cell proliferation and EMT in PTC samples (Figs. 2D, E). CCK8 and EdU assays were carried out to examine cell proliferation in the different groups. The results showed that, compared with the control treatment, ARSI knockdown significantly inhibited PTC cell proliferation (Figs. 2F-H). In addition, the Transwell assay revealed that knockdown of ARSI substantially decreased the migration rate of PTC cells (Figs. 2I, J). To determine whether ARSI expression altered the EMT process in PTC cells, we detected the expression of proteins (N-cadherin and vimentin) associated with EMT by Western blotting. The results indicated that knockdown of ARSI could inhibit the EMT process (Figs. 2K, L). The proliferation-related protein PCNA was also significantly lower in the ARSI-knockdown group than in the control group (Figs. 2M, N) (Figs. S1A, B). These results suggested that ARSI could significantly promote the proliferation, migration and epithelial-mesenchymal transition process of BRAF^V600E^ mutant PTC cells.

### ARSI-mediated gene inhibition triggers ferroptosis in BRAF^V600E^ PTC cells

**Figure 3:**
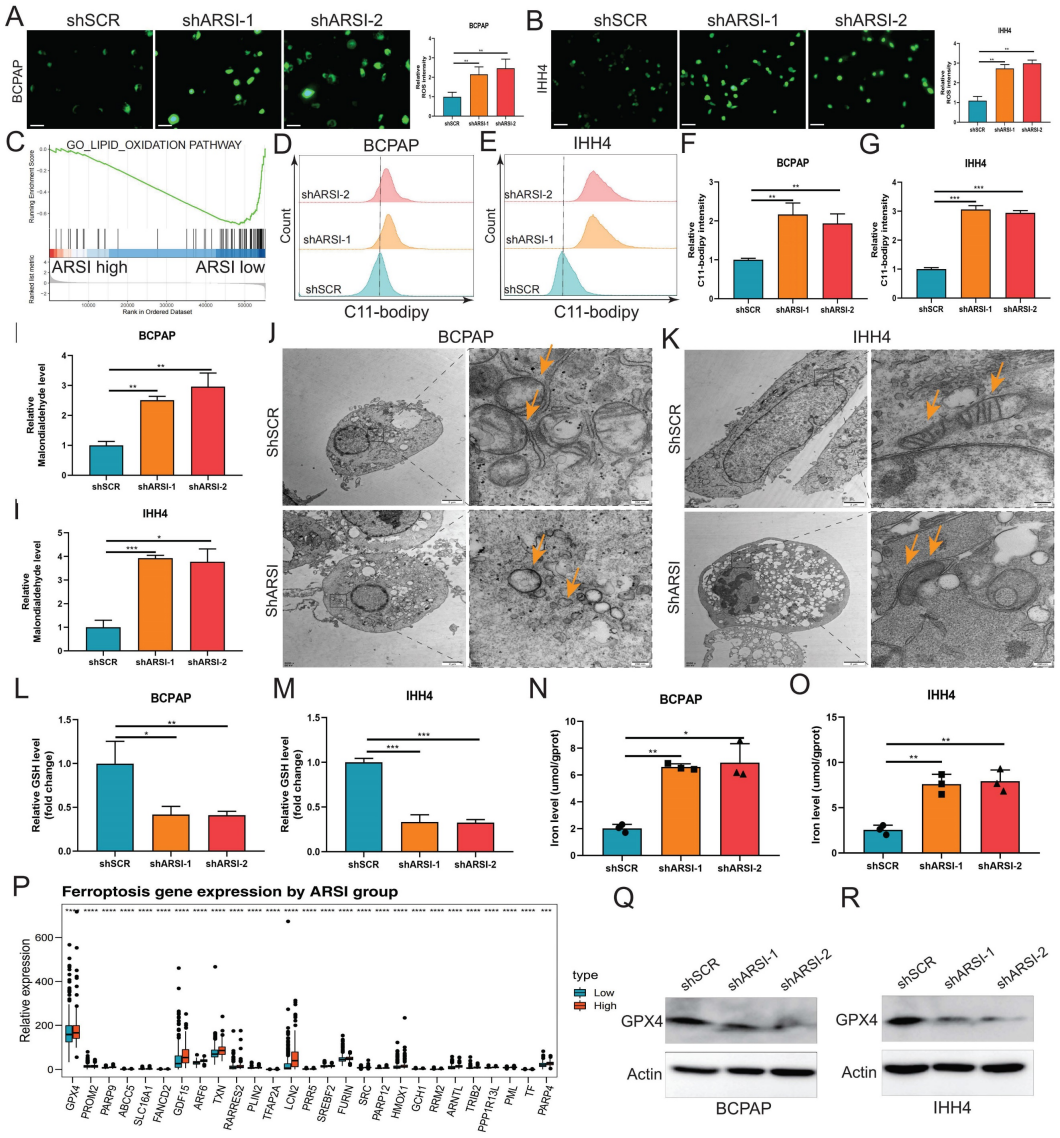
** Gene inhibition of ARSI promotes ferroptosis in BRAF^V600E^ PTC cells. A, B.** Intracellular ROS levels in BCPAP (A) and IHH4 (B) cells were monitored using DCFH-DA fluorescent probes. **C.** GSEA (lipid oxidation pathway) of the ARSI-high and ARSI-low expression subgroups. **D, E**. Lipid ROS were analyzed using C11-BODIPY via flow cytometry. BCPAP (D) and IHH4 (E) cells. **F, G.** Quantitative analysis of lipid ROS levels (n = 3). BCPAP (F) and IHH4 (G) cells. **H, I.** Relative MDA concentrations in PTC cells from different groups. BCPAP (H) and IHH4 (I) cells.** J, K.** Representative TEM images of the mitochondrial structure in BCPAP (J) and IHH4 (K) cells after ARSI knockdown. **L, M.** Relative GSH concentration.** N, O.** Relative iron levels in PTC cells from different groups.** P.** Relative mRNA expression of ferroptosis-related genes. **Q, R.** Western blotting was used to detect the expression of ferroptosis-related proteins. BCPAP (Q) and IHH4 (R) cells. MDA, malondialdehyde; GSH, glutathione; The data are expressed as the mean±SE; * p < 0.05, ** p < 0.01, *** p < 0.001.

Ferroptosis is a new cellular death phenotype distinct from other cell death processes (e.g., apoptosis, autophagy, pyroptosis) and is characterized by an iron dependent, lipid peroxide-driven mechanism^27^. We further explored the role of ARSI in mediating ferroptosis in PTC cells. The detection of intracellular ROS by the fluorescent dye DCFH-DA showed that ROS levels were reduced after ARSI-mediated knockdown in BCPAP and IHH4 cells (Figs. 3A, B). GSEA revealed that the lipid oxidation pathway was enriched in the ARSI-high-expression group (Fig. 3C). The flow cytometry results for detecting lipid ROS indicated that ARSI knockdown increased lipid ROS accumulation in PTC cells (Figs. 3D-G). ARSI knockdown also increased the content of MDA (malondialdehyde), the most common byproduct of lipid peroxidation (Figs. 3H-I). As presented in Figures 3J and K, compared with control groups, PTC cells after ARSI knockdown showed smaller, crumpled, and fractured mitochondria, and the cristae of mitochondria disappeared (Figs. 3J, K). GSH and iron levels were markedly decreased with ARSI inhibition in PTC cells (Figs. 3L-O). Additionally, ARSI inhibition decreased the expression of the ferroptosis-associated protein GPX4 in BRAF^V600E^ PTC cells (Figs. 3Q, R). In conclusion, these results indicate that the inhibition of ARSI suppresses the resistance of BRAF^V600E^ PTC cells to cell death by enhancing their sensitivity to ferroptosis.

### ARSI ablation blocked the growth and metastasis of BRAF^V600E^ PTC cells *in vivo*

**Figure 4:**
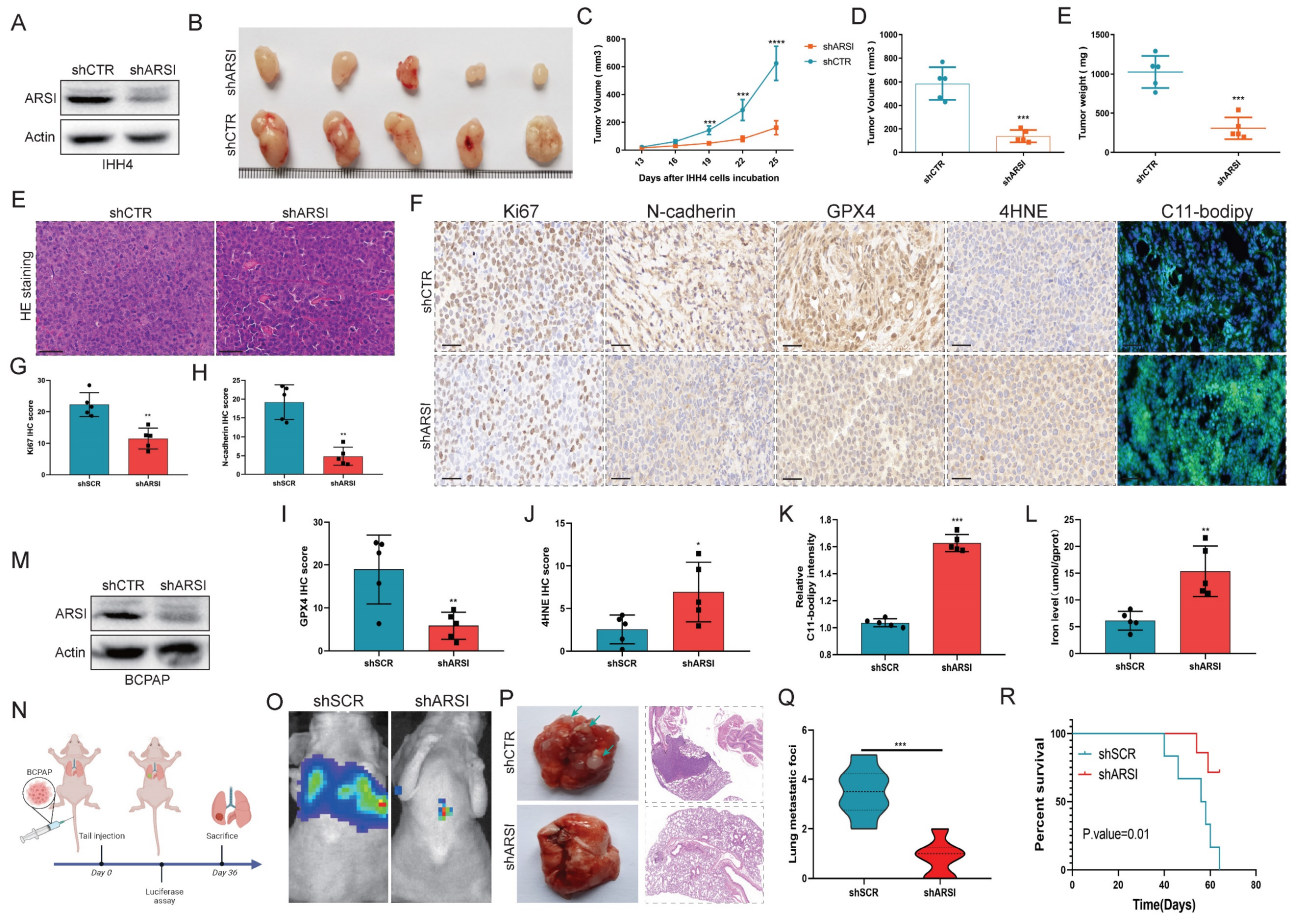
** ARSI ablation blocked the growth and metastasis of BRAF^V600E^ PTC cells *in vivo*. A.** Verification of the knockdown efficiency of ARSI in IHH4 cells. **B**. Representative images of tumors following subcutaneous injection of BRAF^V600E^ PTC cells in nude mice. **C-E.** Tumor volume and weight of xenograft tumors in the two groups.** F.** Representative images of HE staining of xenograft tumors. **F-K**. Representative images and quantification of IHC staining (for Ki67, N-cadherin, GPX4, and 4HNE) and IF staining with the C11-BODIPY probe for solid tumors. **L.** Iron level of solid tumors. **M**. Verification of the knockdown efficiency of ARSI in BCPAP cells. **N**. Schematic diagram of the experimental design. **O**. Images of the luciferase signal in nude mice inoculated with luciferase-labeled BCPAP cells stably transduced with ARSI shRNA or scrambled shRNA. **P**. Lung tumor metastasis and lung histology stained with hematoxylin and eosin in the two groups. **Q.** Quantification of the number of lung metastases. **R.** Kaplan-Meier survival curves for mice with ARSI knockdown versus control cells. The data are expressed as the mean±SE; * p < 0.05, ** p < 0.01, and *** p < 0.001.

To further demonstrate the effect of ARSI expression on PTC progression, IHH4 cells stably transfected with ARSI lentivirus were subcutaneously injected into the backs of nude mice. The silencing efficiency of ARSI was evaluated through protein expression analysis of IHH4 cells (Fig. 4A). As shown in Figure 4B, tumor growth was strongly inhibited by ARSI knockdown. Similarly, the tumor volume and tumor weight in the ARSI-knockdown group were lower than those in the control group (Figs. 4C-E). Moreover, compared with those in the control group, the protein expression of Ki-67, N-cadherin, GPX4 and 4HNE in the ARSI-knockdown tumors was dramatically lower (Figs. 4F-J). C11-BODIPY staining also revealed increased lipid peroxidation after ARSI knockdown (Figs. 4F, K). We next validated the effect of ARSI on metastasis in a xenograft model. Mice were injected with luciferase-expressing BCPAP cells through the tail vein (Fig. 4N). We observed significantly reduced fluorescence intensity in mice in the ARSI-knockdown group compared with that in the scramble group, indicating that metastasis was suppressed in the ARSI-knockdown group (Fig. 4O). Significantly fewer visible surface nodules were observed in the ARSI-knockdown group than in the scramble group (Figs. 4P, Q). In addition, mice in the ARSI-knockdown group tolerated tumors better and survived longer than did the control mice (Fig. 4R).

### Knockdown of ARSI sensitizes BRAF^V600E^ PTC cells to sorafenib via ferroptosis induction

**Figure 5:**
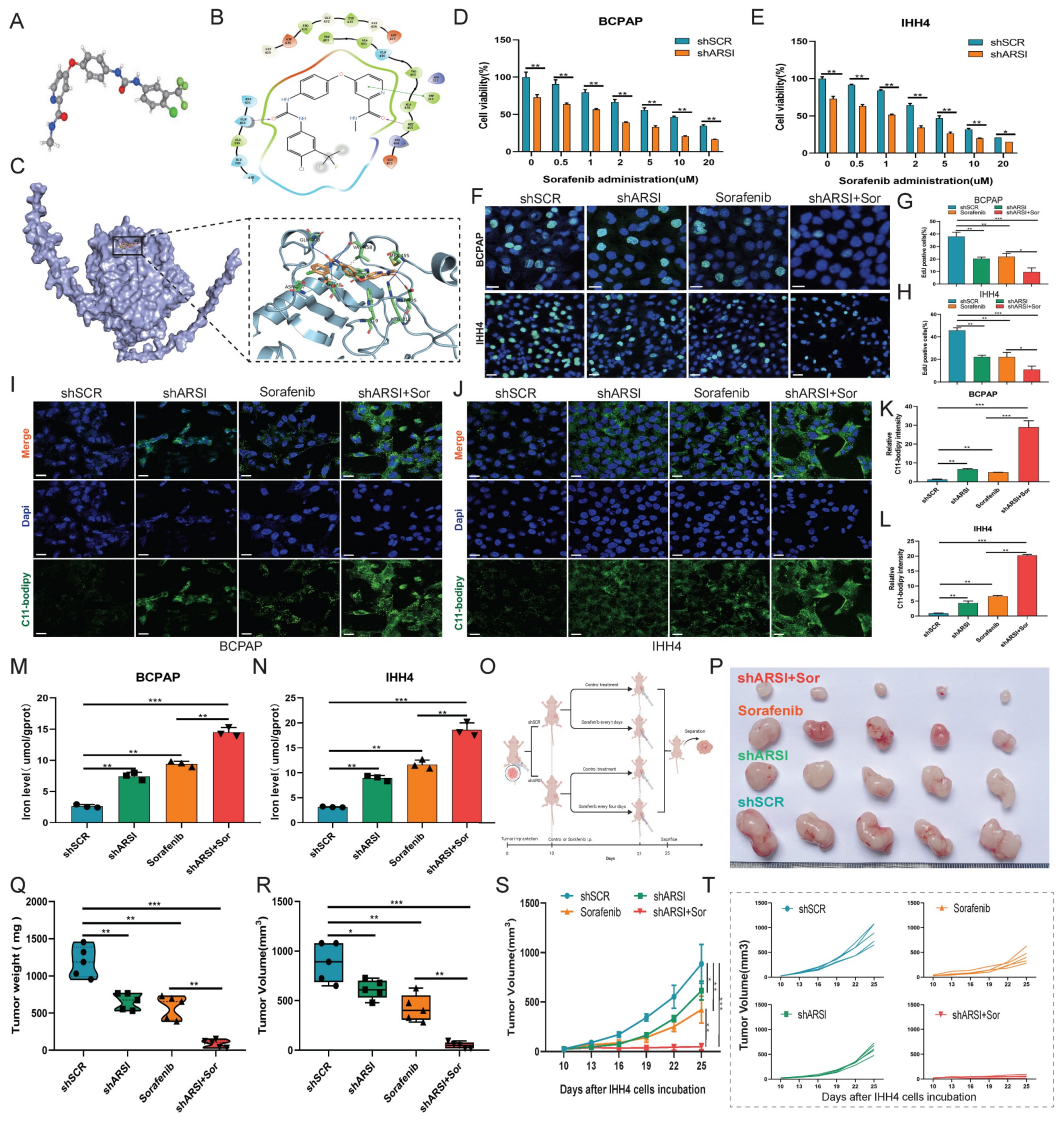
** ARSI knockdown increases the sensitivity of BRAF^V600E^ PTC cells to sorafenib *in vitro* and *in vivo*. A-C.** Molecular formula of sorafenib and ARSI and molecular docking between sorafenib and ARSI. **D, E.** CCK8 assay to analyze the viability of BRAF^V600E^ PTC cells inhibited by ARSI following treatment with different concentrations of sorafenib. **F-H.** Representative images and quantification analysis of the EdU assay results for the indicated groups. **I-L.** IF staining with the C11-BODIPY probe and quantification of C11-BODIPY in transfected BRAF^V600E^ PTC cells after sorafenib treatment. **M, N.** Cellular iron levels in transfected cells after sorafenib treatment. **O**. Schematic diagram of the experimental design. **P**. Representative image of tumors from each group of mice. N = 5 in each group. **Q-T**. Relative tumor weight and volume over time in each group of mice. The data are expressed as the mean±SE; * p < 0.05, ** p < 0.01, and *** p < 0.001.

As an anticancer drug, sorafenib has been shown to induce ferroptosis in cancer cells. We treated PTC cells with various concentrations of sorafenib (0, 0.5, 1, 2, 5, 10 , and 20 μM) to induce ferroptosis. ARSI knockdown increased the toxicity of sorafenib at different concentrations (Figs. 5D, E). EdU incorporation assays revealed that ARSI knockdown inhibited cell division (Figs. 5F-H). C11-BODIPY staining assays showed that ARSI knockdown promoted the increase in oxidized lipids induced by sorafenib (Figs. 5I-L). In addition, the content of iron ions increased in the ARSI shRNA group after treatment with sorafenib (Figs. 5M, N). Next, we implanted nontransfected or transfected BCPAP cells (vector, ARSI-shRNA) into nude mice, and 10 days later, saline or sorafenib was injected intraperitoneally every three days for 15 days (Fig. 5O). Compared to those in saline-treated mice, the tumors in sorafenib-injected mice were consistently smaller (Figs. 5P-T), indicating that sorafenib suppresses tumor growth *in vivo*. The tumors in the sorafenib-injected mice implanted with ARSI shRNA-transfected cells (sorafenib+shARSI) were significantly smaller than those in the sorafenib-injected mice bearing vector-transfected cells (sorafenib+shSCR), suggesting that knockdown of ARSI enhances the antitumor effect of sorafenib (Figs. 5P-T). In addition, there was no significant change in body weight (Fig. S2A) or obvious signs of toxicity. Histological analysis of lung, heart, liver, kidney, and spleen tissues further confirmed that there was no obvious toxicity (Fig. S2B). Thus, these results suggested that ARSI ablation-mediated ferroptosis plays an important role in the inhibitory effect of sorafenib on BRAF^V600E^ PTC cells.

### ARSI enhanced STAT3 activation and promoted the transcription of EREG in BRAF^V600E^ PTC cells

**Figure 6:**
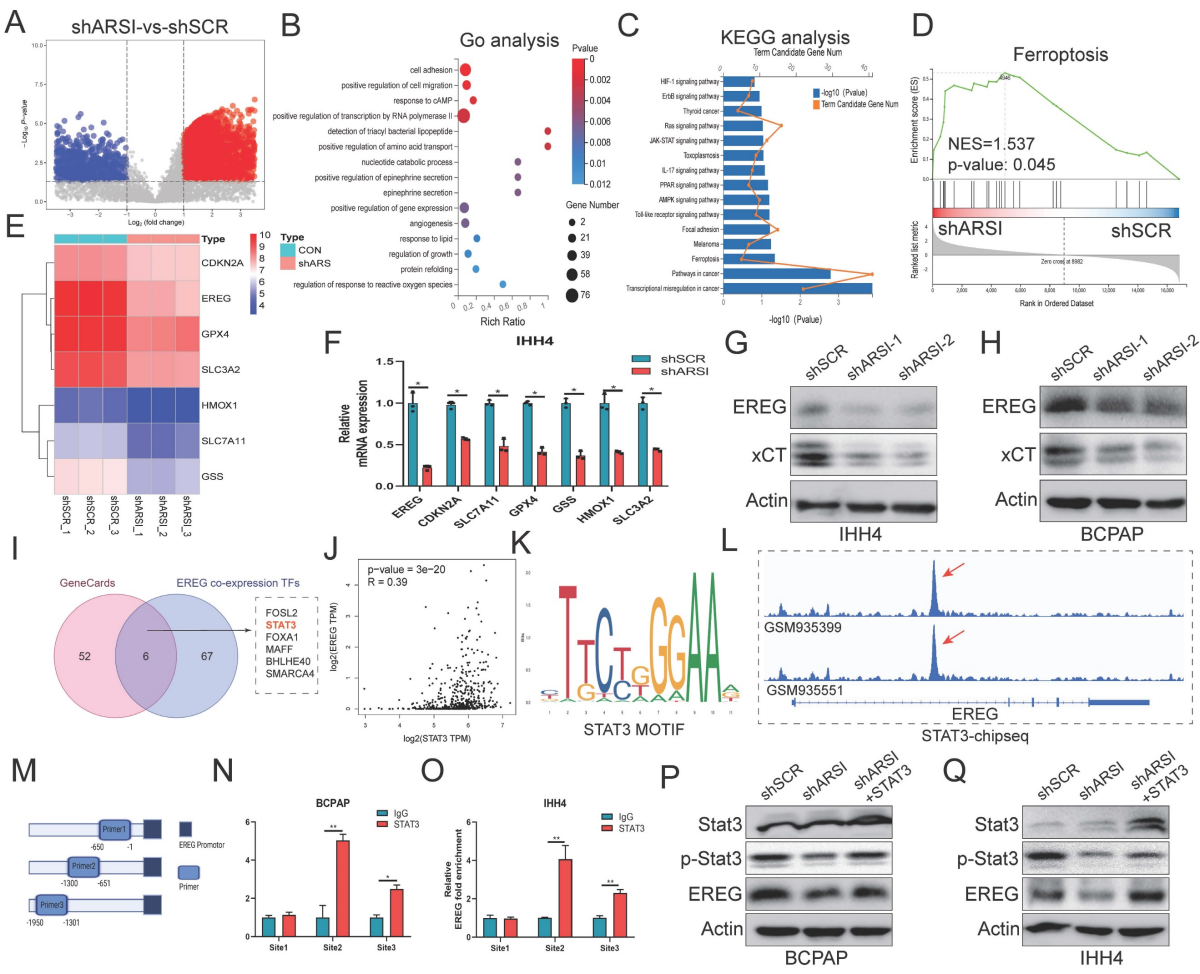
** ARSI enhanced STAT3 activation and stimulated the transcription of EREG in BRAF^V600E^ PTC cells. A.** The analysis of DEGs in ARSI-knockdown IHH4 cells is shown using a volcano plot. **B**. The 15 significantly enriched Gene Ontology BPs of the DEGs. **C**. The 15 significantly enriched KEGG pathways of the DEGs. **D**. GSEA of genes enriched in ferroptosis in the high-ARSI-knockdown group. **E**. Heatmap of ferroptosis-related DEGs in ARSI-knockdown IHH4 cells. **F**. The relative mRNA expression of ferroptosis-related DEGs in IHH4 cells stably transduced with ARSI shRNA or scrambled shRNA. **G, H.** Representative immunoblot showing EREG and xCT expression. **I.** Venn diagram of 6 hub TFs positively related to EREG expression and predicted to bind to the promoter of EREG. **J**. Coexpression analysis of EREG and STAT3 in PTC tissues from the TCGA database. **K**. STAT3 binding motif analysis using JASPAR. **L**. A screenshot of STAT3 and ChIP-seq signals at the EREG locus. **M**. Schematic representation of the EREG (-2000/-1) promoter region. **N, O**. ChIP analysis of STAT3 binding to the EREG promoter in PTC cells using primers as indicated on the x-axis. **P, Q.** Representative immunoblots showing EREG, STAT3 and p-STAT3 expression. The data are expressed as the mean±SE, * p < 0.05.

To further elucidate the underlying mechanisms of ARSIs in regulating BRAF^V600E^ PTC progression, IHH4 cells were analyzed using RNA-seq, and DEGs were obtained from mRNA expression profiling (Fig. 6A). The ten BP categories with the smallest adjusted p values are shown in Figure 6B. KEGG analysis revealed that the DEGs were enriched in the ferroptosis and transcriptional misregulation pathways in cancer (Fig. 6C). Consistently, the results of GSEA showed that ferroptosis was one of the hallmarks of the DEGs (Fig. 6D). The significant ferroptosis-related DEGs, including the best-characterized ferroptosis genes GPX4 and SLC7A11, are shown in Figure 6E. Strikingly, the list also included epiregulin (EREG), an ErbB family ligand encoded by the EREG gene. The hub ferroptosis-related regulators were further confirmed in ARSI-knockdown IHH4 cells, and EREG exhibited the most significant difference (Fig. 6F). The expression of both EREG and xCT was downregulated in ARSI-knockdown PTC cells (Figs. 6G, H). As transcription factor (TF) dysregulation is associated with tumor progression, we analyzed the TFs predicted to bind to the promoter of EREG via the Cistrome Data Browser. Six candidate TFs were screened through the intersection of the GeneCards and EREG coexpression data (Fig. 6I). Coexpression analysis revealed that the mRNA expression of ARSI was most strongly correlated with that of STAT3 compared to that of the other 5 TFs in PTC (Fig. 6J). JASPAR database analysis revealed the STAT3 binding motif in the EREG gene (Fig. 6K). ChIP-seq data were analyzed to determine the molecular mechanism responsible for the STAT3-induced increase in LIP, and the results revealed that STAT3 was enriched in the promoter region of the EREG gene (Fig. 6L). A ChIP‒PCR assay was used to detect the interaction of STAT3 with EREG promoter regions (Figs. 6M-O). We then performed rescue experiments by cotransfecting BRAF^V600E^ PTC cells with ARSI shRNA and the STAT3 plasmid. As expected, transfection of the STAT3 plasmid into ARSI-knockdown cells rescued the decrease in EREG protein levels caused by ARSI knockdown (Fig. 6P, Q). Taken together, these results indicated that STAT3 increased EREG expression through transcriptional activation in BRAF^V600E^ PTC cells.

### EREG mediates ARSI-driven ferroptosis resistance and malignant phenotypes in BRAF^V600E^ PTC cells

**Figure 7:**
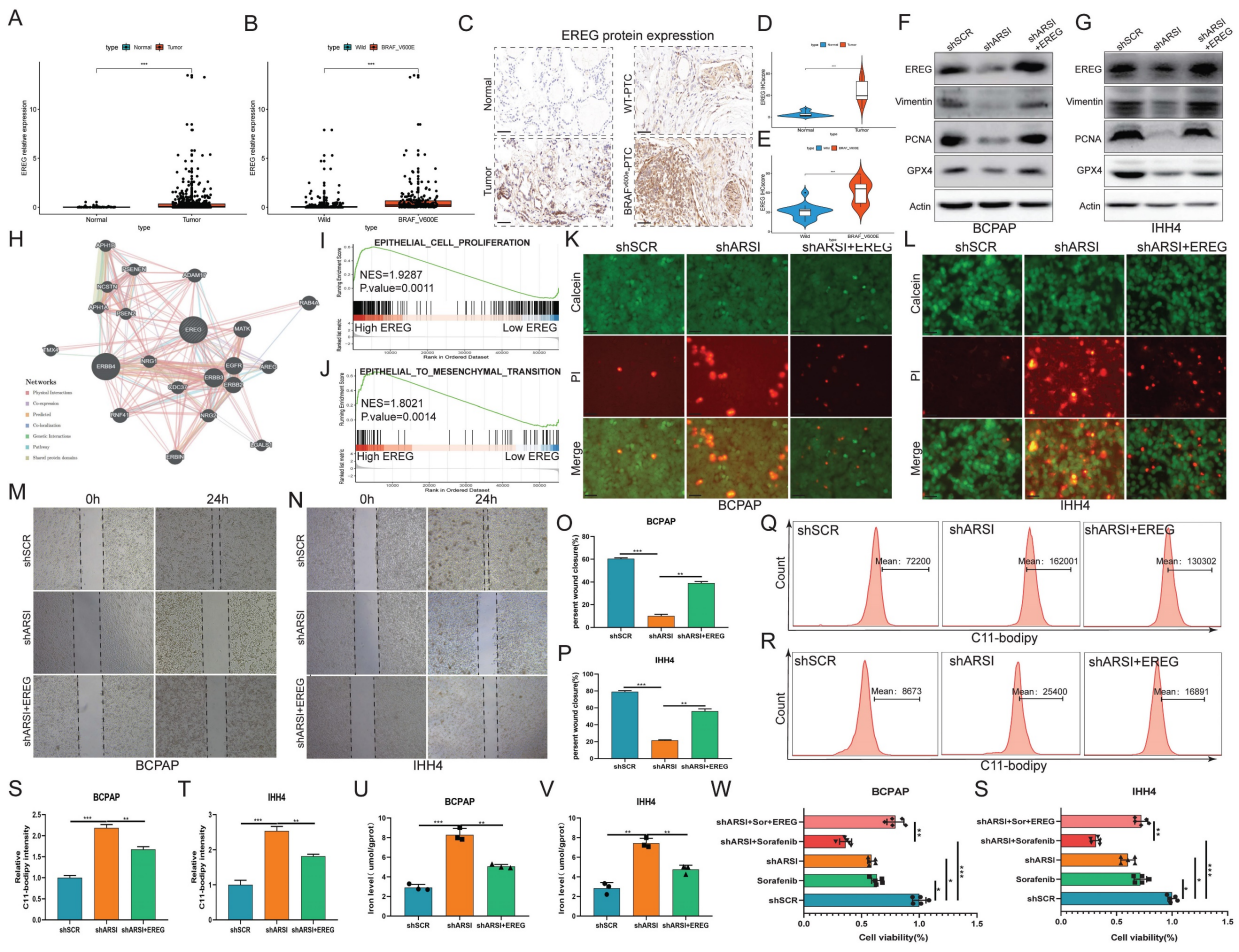
** EREG mediated ARSI-driven anti-ferroptosis and malignant effects on BRAF^V600E^ PTC cells. A.** EREG levels in PTC and noncancer tissues.** B.** EREG expression based on the BRAF mutation genotype in the THCA cohort. **C-E**. Immunohistochemical staining and IHC score of the EREG protein level based on the BRAF mutation status in PTC samples. **F, G.** Representative immunoblots of PCNA, EREG, GPX4 and Vimentin. **H.** PPI network of EREGs and related hub genes according to the GeneMANIA database. **I, J.** GSEA of genes enriched in cell proliferation and epithelial-mesenchymal transition in the high-EREG expression group.** K, L.** Fluorescence images of BRAF^V600E^ PTC cells; dead cells were labeled with PI (red), and live cells were labeled with calcein AM (green). Scale bars: 50 µm. **M-P.** Cell migration was determined by wound healing assay. **Q-T.** Lipid ROS were analyzed using C11-BODIPY via flow cytometry. **U, V.** Relative iron levels in BRAF^V600E^ PTC cells from different groups. **W, S.** The CCK8 assay was used to detect BRAF^V600E^ PTC cell proliferation. The data are expressed as the mean±SE; * p < 0.05, ** p < 0.01, *** p < 0.001.

To further explore the potential oncogenic role of EREG in PTC patients with the BRAF^V600E^ mutation, we compared the expression of EGERs in PTC patients from the TCGA database. As expected, the EGER was significantly increased in patients with the BRAF mutation (Figs. 7A, B). IHC analysis of clinical human BRAF^V600E^ PTC and corresponding BRAF^WT^-PTC samples revealed that EGER is highly expressed in BRAF-mutant tumor tissue (Figs. 7C-E). Transfection of the EREG plasmid into ARSI-knockdown cells mitigated the decreases in the protein levels of Vimentin, PCNA and GPX4 caused by ARSI knockdown (Figs. 7F, G). The PPI network of EREG and related hub genes was constructed via the GeneMANIA database (Fig. 7H). GSEA also revealed that the signatures associated with cell proliferation and EMT were mediated by high expression of EREG (Figs. 7I, J). Consistently, EGER overexpression also mitigated the decrease in the number of dead cells (Figs. 7K, L) and the invasion of tumor cells (Figs. 7M-P) in the ARSI-knockdown group. EREG overexpression also caused an increase in lipid peroxidation levels, as determined using the C11-BODIPY probe (Figs. 7Q-T). Similarly, an increase in Fe^2+^ was also observed via flow cytometry analysis (Figs. 7U, V). The effects of EREG overexpression on the proliferation of PTC cells were also examined (Figs. 7W, S). Taken together, the upregulation of EREG through STAT3 mediated ARSI-driven ferroptosis resistance and malignant phenotypes in PTC cells.

### Construction of a prognostic signature and diagnostic model based on the ARSI and EREG signature in PTC patients

**Figure 8:**
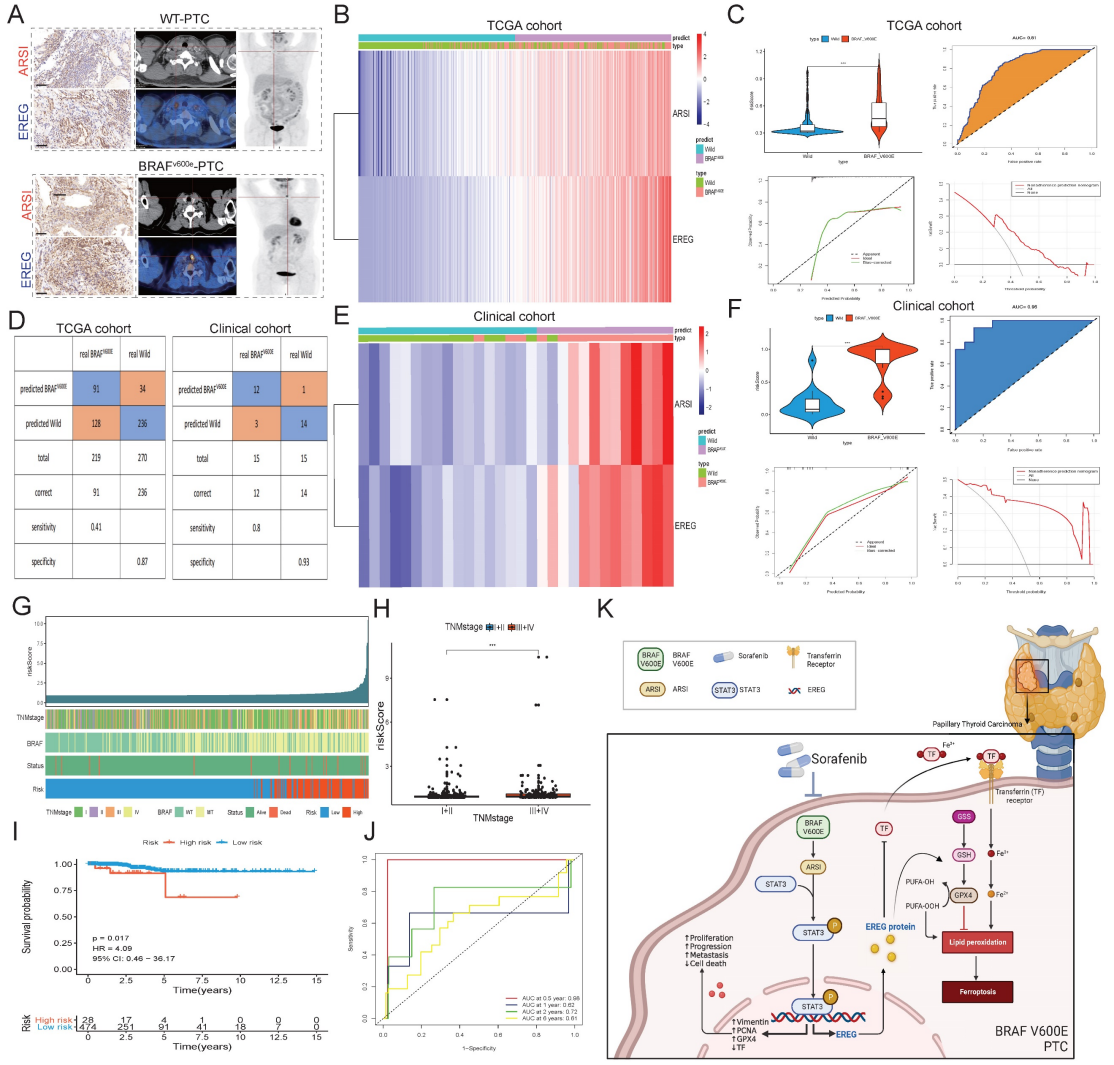
** Construction of a prognostic signature and diagnostic model based on the ARSI and EREG signatures in the PTC cohort. A.** Representative IHC and PET-CT scans of PTC patients. **B.** Heatmaps of ARSI and EREG expression between BRAF^V600E^ and BRAF^WT^ tissues in the TCGA cohort. **C.** Distribution of risk scores for BRAF^V600E^ and BRAF^WT^ PTC patients in the TCGA cohort. **D.** Diagnostic model for distinguishing BRAF^V600E^ and BRAF^WT^ patients from the TCGA and clinical cohorts. **E.** Heatmaps of ARSI and EREG expression between BRAF^V600E^ and BRAF^WT^ tissues in clinical cohorts. **F.** Distribution of risk scores of PTC patients in the clinical cohort. **G.** Correlations between the risk score and clinical features (including survival status, BRAF mutation status and TNM stage) in PTC patients. **H.** Correlation between the risk score and TNM stage of PTC patients. **I.** K‒M curves of patients in the high- and low-risk groups. **J.** Time-dependent ROC analysis for OS prediction in the TCGA-THCA cohort. **K.** Schematic of the mechanism by which BRAF-activated ARSI suppressed EREG-mediated ferroptosis to promote BRAF^V600E^ (mutant) papillary thyroid carcinoma progression and sorafenib resistance.

As both EREG and ARSI exhibited high expression in BRAF^V600E^ cells (Fig. 8A), we constructed a prognostic signature based on ARSI and EREG expression through multivariate Cox regression (Fig. 8B). In the PTC cohort from the TCGA cohort, the risk score of BRAF^V600E^ patients significantly exceeded that of BRAF^WT^ patients (Fig. 8C), with the prognostic signature yielding an AUC of 0.81, indicating substantial predictive value. Moreover, the risk score of PTC tumor tissues was significantly greater than that of normal tissues, with an AUC of 0.73, further underscoring its predictive utility (Figs. S3A-D). The prognostic signature was applied to the clinical cohort for further validation of the prediction ability, and the AUC of the prognostic signature reached 0.96 (Figs. 8E-F). We then applied a stepwise logistic regression method to establish a diagnostic model that integrated these two genes to distinguish between BRAF^V600E^ and BRAF^WT^ patients. The diagnostic model was used to identify BRAF^V600E^ patients in the TCGA cohort with a specificity of 87% and in the clinical cohort with a specificity of 93% (Fig. 8D). PTC patients were classified into high-risk and low-risk groups, and the high-risk group was positively associated with TNM stage and the presence of BRAF mutation (Figs. 8G, H). Moreover, patients in the high-risk group had a significantly shorter OS than did those in the low-risk group (Figs. 8I-J). Thus, these results indicated that ARSI and EREG could also be applied in clinical practice and could provide new options for the treatment and management of BRAF^V600E^ patients.

## Discussion

Papillary thyroid carcinoma, accounting for approximately 90% of thyroid cancer cases, is characterized by increased capsular invasion propensity, organ compression, and a high incidence of lymph node dissemination^28^, ^29^. Despite its prevalence, the etiology and molecular pathogenesis of PTC have not been fully elucidated. The B-type RAF kinase BRAF (V600E) mutation has been identified as a key initiator of PTC carcinogenesis^30^. A comprehensive understanding of the molecular mechanisms underlying PTC pathogenesis is imperative for the development of detection biomarkers and effective therapeutic strategies. Our findings underscore the complex interplay between ARSI, EREG, and ferroptosis in PTC progression and highlight a potential therapeutic strategy for BRAF^V600E^ PTC patients.

Arylsulfatase I (ARSI), a member of the sulfatase gene family, has garnered increasing attention in cancer research due to its diverse roles in tumorigenesis and progression^31^, ^32^. While the primary function of ARSIs involves the hydrolysis of sulfate esters in the extracellular environment, their dysregulation has been implicated in various malignancies, including PTC. In our study, we observed significant upregulation of ARSI expression in BRAF^V600E^ PTC patients, indicating its potential as a biomarker for disease prognosis and therapeutic targeting. Similar upregulation of sulfatases has been reported in other cancers, such as breast and prostate cancer, highlighting a potential common mechanism in tumor progression. However, our study uniquely identifies ARSI as a potential prognostic marker specifically in the context of BRAFV600E PTC, differentiating it from its role in other malignancies.

The associations between ARSI expression and clinicopathological features, such as tumor size, lymph node metastasis, and advanced TNM stage, underscore its relevance to PTC aggressiveness and disease progression. The functional characterization of ARSIs in BRAF-mutant PTC cells revealed their role in promoting cell proliferation, migration, and EMT, thereby contributing to the acquisition of aggressive phenotypes. Notably, ARSI knockdown induced ferroptosis, a novel form of programmed cell death characterized by iron-dependent lipid peroxidation, suggesting a potential therapeutic strategy for inhibiting PTC progression. Further elucidation of the molecular mechanisms underlying the oncogenic effects of ARSI is warranted. ARSI may modulate extracellular matrix remodeling, cell signaling pathways, and immune responses in the tumor microenvironment, thereby influencing tumor growth and metastasis. Additionally, exploring the interactions of ARSI with other proteins and signaling pathways could unveil novel therapeutic targets for PTC treatment.

Epiregulin (EREG), a member of the epidermal growth factor (EGF) family, plays crucial roles in cell proliferation, survival, and differentiation, making it an attractive target for cancer therapy^33^, ^34^. In our study, EREG emerged as a potential mediator of ARSI-driven ferroptosis resistance and malignant phenotypes in PTC cells, particularly in the context of the BRAF^V600E^ mutation. The regulatory axis involving STAT3-mediated transcriptional activation of EREG is a potential therapeutic target for PTC treatment. Targeting EREG signaling pathways or upstream regulators, such as STAT3, could offer promising avenues for inhibiting PTC growth and metastasis. Our findings of EREG involvement in ARSI-driven ferroptosis resistance extend previous research that has highlighted EREG's role in other cancer types. For instance, EREG has been shown to promote resistance to targeted therapies in non-small cell lung cancer. The unique aspect of our study is its focus on the EREG/STAT3 axis in BRAFV600E PTC, a specific regulatory pathway that has not been extensively studied in this context.

Ferroptosis is a form of regulated cell death characterized by iron-dependent lipid peroxidation and is distinct from other types of cell death, such as apoptosis, autophagy, and necrosis^18^. Emerging evidence suggests that dysregulation of ferroptosis plays a critical role in cancer progression and therapeutic resistance, making it an attractive target for cancer therapy^35^, ^36^. In our study, we investigated the role of ferroptosis in PTC and its modulation by ARSI and EREG. Our findings demonstrated that knockdown of ARSI induces ferroptosis in BRAF-mutant PTC cells, suggesting a novel therapeutic strategy for inhibiting PTC progression. Sorafenib, a multikinase inhibitor, has been shown to induce ferroptosis in cancer cells, suggesting that this is a promising therapeutic strategy for overcoming drug resistance in PTC^37^, ^38^. Our findings suggest that ARSI inhibition enhances the efficacy of sorafenib by promoting ferroptosis in PTC cells, providing a rationale for combination therapy in PTC patients with the BRAF mutation.

Our research provides valuable insights into the role of ARSIs and EREGs in BRAF^V600E^ PTC; however, there are several limitations that should be considered. While the study identified ARSI as a potential prognostic marker and therapeutic target, the findings primarily rely on preclinical data and retrospective analyses. Prospective clinical validation in independent cohorts is necessary to confirm the prognostic value of ARSI and evaluate its potential as a therapeutic target in BRAF^V600E^ PTC patients. Future clinical studies with long-term follow-up should be analyzed to assess the prognostic value of ARSI and EREG in PTC. Moreover, *in vitro* experiments provided valuable mechanistic insights into the role of ARSIs in PTC cells. However, the translation of these findings to the complex *in vivo* tumor microenvironment should be interpreted cautiously.

In conclusion, our findings highlight the significance of EREG in mediating ARSI-driven oncogenic effects in PTC cells and underscore its potential as a therapeutic target for PTC treatment. Further research into the molecular mechanisms regulating the ARSI/EREG axis and its interactions with other signaling pathways could lead to the development of novel targeted therapies for BRAF^V600E^ PTC patients, ultimately improving clinical outcomes and patient survival.

## Supplementary Material

Supplementary figures and tables.
