# Supplementary material for: BRAF-activated ARSI suppressed EREG-mediated ferroptosis to promote BRAFV600E (mutant) papillary thyroid carcinoma progression and sorafenib resistance
Source: Int J Biol Sci. 2025 Jan 1;21(1):128–42. doi: 10.7150/ijbs.99423 (PMC11667812; doi:10.7150/ijbs.99423)
Supplement: Supplementary file 1 — Supplementary figures and tables. [file ijbsv21p0128s1.pdf]

BRAF-activated ARSI suppressed EREG-mediated ferroptosis to promote BRAF^{V600E} (mutant) papillary thyroid carcinoma progression and sorafenib resistance

Xing Chen[1#*], Xiang Chen[2#], Wenjun Xie[1#], Hua Ge[3], Hongyan He[4], Ailong Zhang[1], Junjie Zheng[1]

1. Department of Thyroid and Hernia Surgery, Fujian Provincial Hospital, Shengli Clinical Medical College of Fujian Medical University, Fuzhou City, Fujian Province 350001, China.
2. Department of Ultrasonography , Fujian Provincial Hospital, Shengli Clinical Medical College of Fujian Medical University, Fuzhou City, Fujian Province 350001, China
3. Department of Nuclear Medicine, Fujian Provincial Hospital, Shengli Clinical Medical College of Fujian Medical University, Fuzhou City, Fujian Province 350001, China.
4. Department of Pathology, Fujian Provincial Hospital , Shengli Clinical Medical College of Fujian Medical University, Fuzhou City, Fujian Province 350001, China.

# These authors contributed equally.

*Correspondence: Xing Chen, jianyuhaohai2022@163.com

Supplementary Table S1: qRT-PCR primers used in the study

Name	Sequence
β-actin	Forward: 5′-AGTTGCGTTACACCCTTTCTTG-3′<br>Reverse: 5′-CACCTTCACCGTTCCAGTTTT-3′
EREG	Forward: 5'-ATCCTGGCATGTGCTAGGGT-3'<br>Reverse: 5'-GTGCTCCAGAGGTCAGCCAT-3'
SLC7A11	Forward: 5′-TATCCCTGGCATTTGGAC-3′<br>Reverse: 5′-TCACTACAGTTATGCCCACAG-3′
GPX4	Forward: 5′-TGGGAAATGCCATCAAGTGG-3′<br>Reverse: 5′-GGTCCTTCTCTATCACCAGGGG-3′
GSS	Forward: 5′-ACAGGATGACTTTACCGCTC-3′<br>Reverse: 5′-AGCGGTAAAGTCATCCTGTT-3′
HMOX1	Forward: 5′-TCAGGCAGAGGGTGATAGAAG-3′<br>Reverse: 5′-TTGGTGTCATGGGTCAGC-3′
SLC3A2	Forward: 5′-ACTTGGCTGAGTGGCAGAAT-3′<br>Reverse: 5′-AGATCGCTGGTGGATTCAAG-3′
CDKN2A	Forward: 5′-ATGGAGCCTTCGGCTGACT-3′<br>Reverse: 5′-GTAACTATTCGGTGCGTTGGG-3′

Supplementary Table S2: ChIP-qPCR primers used in the study

Name	Sequence
EREG-1	Forward: 5′-CTCCATCAGCATAGGCAGGAA -3′
	Reverse: 5′-GGTTGCTCAGGGTGACGGACATA-3′
EREG-2	Forward: 5'-TATGTCCGTCACCCTGAGCAACC-3'
	Reverse: 5'-CGGCGATGGGAGCGGGCGCT-3'
EREG-3	Forward: 5′-ACGTGTGGCTCAAGTGTCAA-3′
	Reverse: 5′-CACTTCACACC TGCAGTAGTTT-3

Figure S1. Quantification of PCNA expression in BRAF^{V600E} PTC cells with ARSI inhibition.

A-B Quantification of PCNA expression in BCPAP cells (A) and IHH4 cells (B).

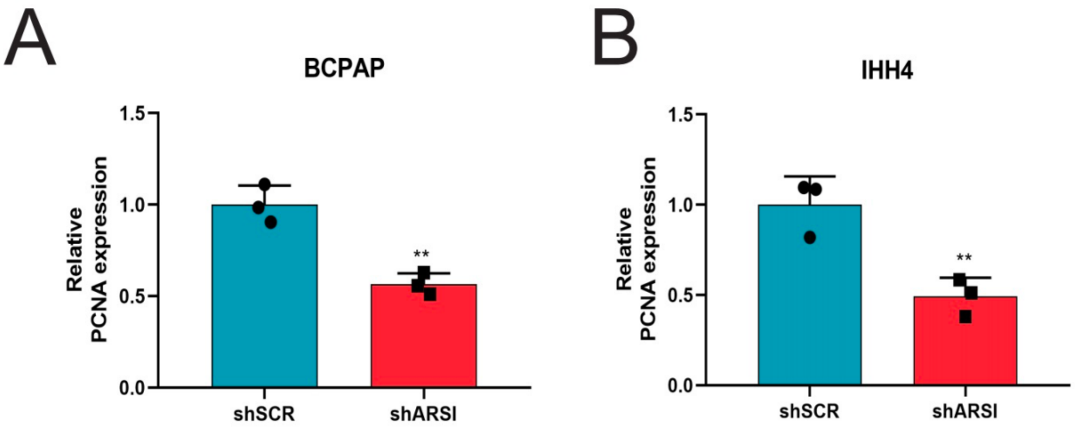

Figure S2. No evident toxicity was observed under the various treatment regimens.

A. Body weight of the mice in each group. B. Histological analysis of tissues from the lung, heart, liver, kidney, and spleen.

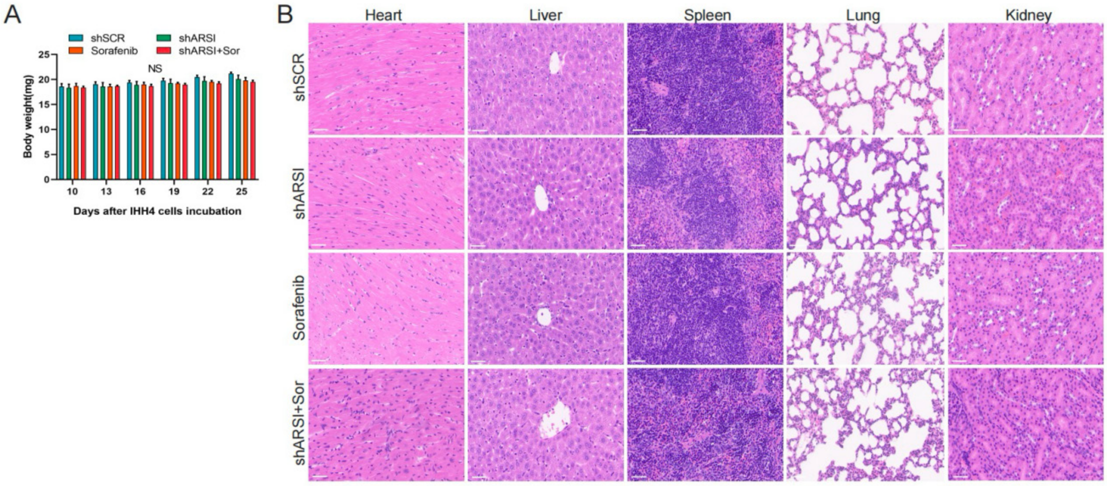

Figure S3. Construction of a prognostic signature and diagnostic model based on ARSI and EREG signatures in PTC samples.

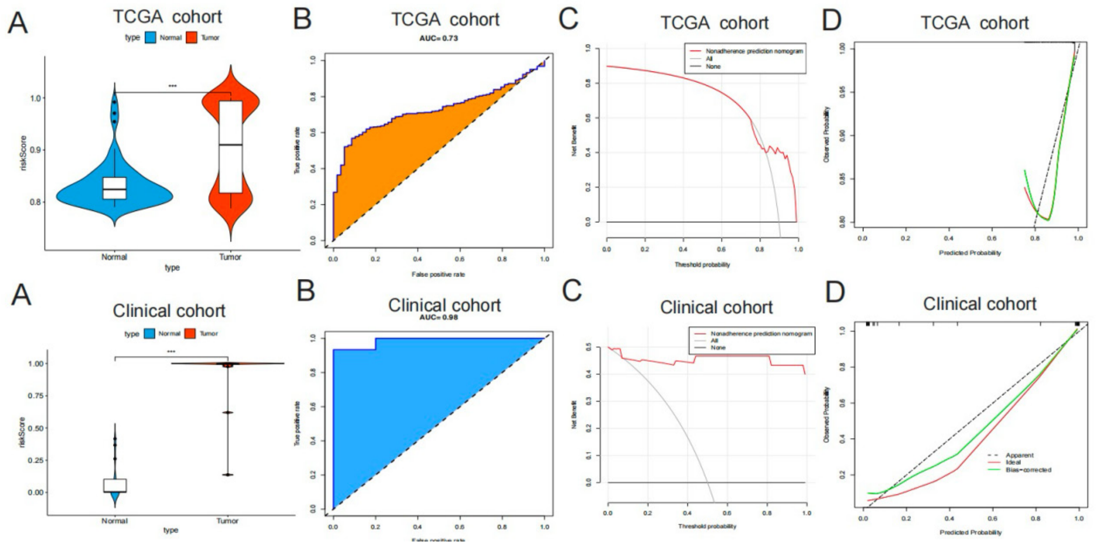

A. Distribution of risk scores in PTC tumor and normal tissues from the TCGA cohort. B-D. ROC curves for the predictive performance of the diagnostic model.
